# Restoration of Spermatogenesis and Male Fertility Using an Androgen Receptor Transgene

**DOI:** 10.1371/journal.pone.0120783

**Published:** 2015-03-24

**Authors:** William H. Walker, Evan Easton, Rebecca S. Moreci, Corey Toocheck, Prashanth Anamthathmakula, Pancharatnam Jeyasuria

**Affiliations:** Center for Research in Reproductive Physiology, Department of Obstetrics, Gynecology and Reproductive Sciences, Magee Women’s Research Institute, University of Pittsburgh, Pittsburgh, Pennsylvania, United States of America; University Hospital of Münster, GERMANY

## Abstract

Androgens signal through the androgen receptor (AR) to regulate male secondary sexual characteristics, reproductive tract development, prostate function, sperm production, bone and muscle mass as well as body hair growth among other functions. We developed a transgenic mouse model in which endogenous AR expression was replaced by a functionally modified AR transgene. A bacterial artificial chromosome (BAC) was constructed containing all AR exons and introns plus 40 kb each of 5' and 3' regulatory sequence. Insertion of an internal ribosome entry site and the EGFP gene 3’ to AR allowed co-expression of AR and EGFP. Pronuclear injection of the BAC resulted in six founder mice that displayed EGFP production in appropriate AR expressing tissues. The six founder mice were mated into a Sertoli cell specific AR knockout (SCARKO) background in which spermatogenesis is blocked at the meiosis stage of germ cell development. The AR-EGFP transgene was expressed in a cyclical manner similar to that of endogenous AR in Sertoli cells and fertility was restored as offspring were produced in the absence of Sertoli cell AR. Thus, the AR-EGFP transgene under the control of AR regulatory elements is capable of rescuing AR function in a cell selective, AR-null background. These initial studies provide proof of principle that a strategy employing the AR-EGFP transgene can be used to understand AR functions. Transgenic mice expressing selective modifications of the AR-EGFP transgene may provide crucial information needed to elicit the molecular mechanisms by which AR acts in the testis and other androgen responsive tissues.

## Introduction

Androgens are a class of steroid hormones that regulate prostate function, bone density, cardiac health, muscle mass, hair growth and fertility. Androgens diffuse through the plasma membrane and act via the intracellular androgen receptor (AR) to alter gene expression and intracellular signaling pathways in target cells. The two major functional androgens in mammals are testosterone and dihydrotestosterone (DHT). Because of the high levels of testosterone produced locally by the Leydig cells within the testis, this form of androgen is the major regulator of testis functions and the male reproductive tract. In most other tissues, the lower concentrations of testosterone present allow DHT to be the major acting androgen because DHT has a 10-fold greater affinity for AR than testosterone.

There are 2 two pathways by which androgens act to regulate cellular function. In the classical pathway, androgen interacts with AR in the cytoplasm that then translocates to the nucleus where it binds androgen response element DNA sequences and directly regulates gene expression. In the non-classical pathway, androgens act via AR, in the cytoplasm, to rapidly activate kinase cascades or alter intracellular [Ca^2+^] levels [1]. The resulting phosphorylation changes alter the activities of target proteins that can cause immediate changes in cellular physiology as well as indirect or delayed effects including altered gene expression. Non-classical AR action has been documented in numerous cell types including skeletal muscle fibers [2], cardiac myocytes [3], neurons [4], prostate cancer cells [5], macrophages and T-cells [6] as well as Sertoli cells (reviewed in [7]).

In males, testosterone is essential for proper sexual differentiation and the maintenance of spermatogenesis, which is the progression of germ cell development into mature sperm [8, 9]. Functional androgen receptor is not expressed in germ cells [10–12]. However, testosterone support for germ cell development occurs via the Leydig, peritubular myoid cells and Sertoli cells that express AR. Sertoli cells are the major transducers of testosterone signals to the adjacent germ cells. Assessments of spermatogenesis after testosterone deprivation studies and examinations of Sertoli cell specific AR knock out (SCARKO) mice have shown that testosterone signaling through the AR in Sertoli cells is required to maintain the blood testis barrier [13, 14] for the completion of meiosis (reviewed in [15]), maintaining the attachment of germ cells to Sertoli cells and the release of mature spermatozoa [16–18].

The molecular mechanisms are beginning to be known by which androgen and AR regulate processes in Sertoli cells that are required to maintain spermatogenesis. For example, there is reduced expression of at least three tight junction components of the BTB (occludin, claudin 11 and claudin 3) in the absence of AR expression in Sertoli cells [13]. AR also is needed for the recycling of BTB proteins during the remodeling of the BTB [19, 20]. Androgen suppression disrupts two of the protein complexes that form the specialized connections (cadherin/cadherin and α6β1-integrin/lamininγ3) between Sertoli cells and the elongated spermatids [21, 22]. In the absence of androgen, there is an alteration in the phosphorylation of proteins including focal adhesion kinase and β-catenin that decrease the integrity of the cadherin/cadherin and α6β1-integrin/lamininγ3intracellular connections leading to premature detachment of elongated spermatids [21, 23].

Studies of SCARKO mice and testosterone depletion experiments have revealed specific processes in Sertoli cells that require testosterone action. However, little information is available regarding the specific functions of classical versus non-classical androgen signaling in regulating critical spermatogenesis processes. An *in vivo* model system in which endogenous AR expression is replaced with specific AR mutants that selectively activate either the classical or non-classical testosterone pathways would provide an essential tool for dissecting the molecular mechanisms by which AR acts. As a first step in achieving this goal, we produced transgenic mouse strains that co-express AR and EGFP from a BAC insert containing the complete mouse genomic AR gene and 40 kb each of 5’ and 3’ flanking sequences. The AR-EGFP transgene is expressed in testosterone responsive tissues and is correctly expressed in AR positive cells of the adult testis. Furthermore, we report the first transgenic rescue of AR function as the AR-EGFP transgene is able to restore complete spermatogenesis and fertility in a SCARKO mouse that lacks endogenous AR expression in Sertoli cells.

## Materials and Methods

### Ethics Statement

All mouse procedures were approved under protocol IS00000296 titled Nongenomic Androgen Signaling in Sertoli Cells by the University of Pittsburgh Institutional Animal Care and Use Committee (IACUC), which operates under protocol A3187-01 from the Association for Assessment and Accreditation of Laboratory Animal Care (AAALAC).

### Construction of a BAC encompassing the AR genomic gene and 40 kb each of 5’ and 3’ flanking regions

**Fig 1:**
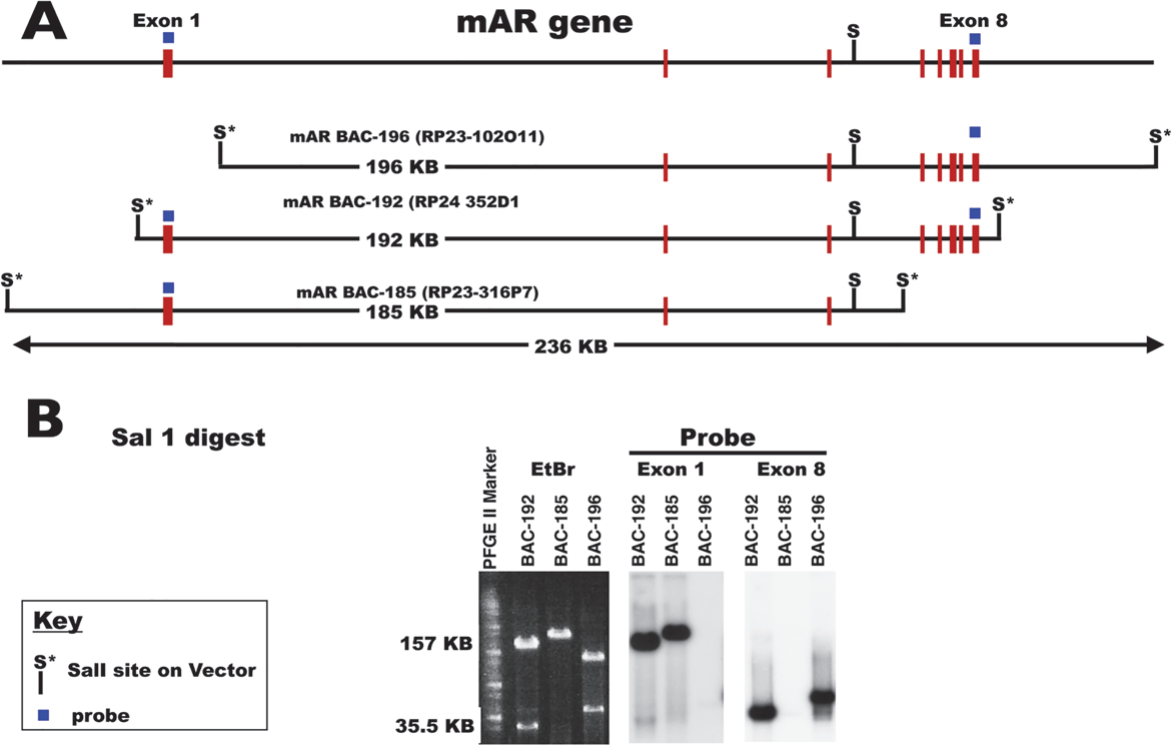
Characterization of three BACs containing parts of the AR gene. (A) Top: a map of the AR gene and flanking region is shown. Below: maps of the BACs mAR BAC-196 (RP23-102011), mAR BAC-192 (RP24-352D1) and mAR BAC-185 (RP23-316P7) are shown. AR exons are denoted by rectangles on the lines. *SalI* restiction sites are denoted with an S. Boxes above exons 1 and 8 denote the probes used for Southern blotting. The BACs contained either extended 5’ sequence, the complete AR gene or extended 3’ sequence, respectively. The BACs were end sequenced to determine their insertion sites. (B) Restriction digests followed by PFGE and Southern blotting were performed to ensure that preperations of the BACs were correct. The *SalI* digested BACs were fractionated by PFGE and subjected to Southern blotting using ^32^P random primed labelled AR exon 1 and AR exon 8 probes. mAR BAC-192 was positive for both probes while mAR BAC-196 only probed positive for exon 8 and mAR BAC-185 probed positive only for exon1.

Three BACs containing all or parts of the gene encoding AR were obtained from the BACPAC Resource Center (BPRC) at Children's Hospital Oakland Research Institute (CHORI, Oakland, CA). One BAC [mAR BAC-192 (RP24-352D1)] contains the entire gene (all exons) for AR. The other two BACs contain either 5’ [mAR BAC-185 (RP23-316P7)] or 3’ [mAR BAC-196 (RP23-102O11)] exons of the AR gene and either extensive 5’ or 3’ flanking regions (Fig. 1A). The BACs containing the AR genomic sequence were end sequenced to establish the positions of the inserts. Pulse field gel electrophoresis (PFGE) in combination with Southern blotting was used to verify the inserts by comparisons of restriction analysis of the BACs with known genomic sequence from the mouse genome project (Fig. 1B).

**Fig 2:**
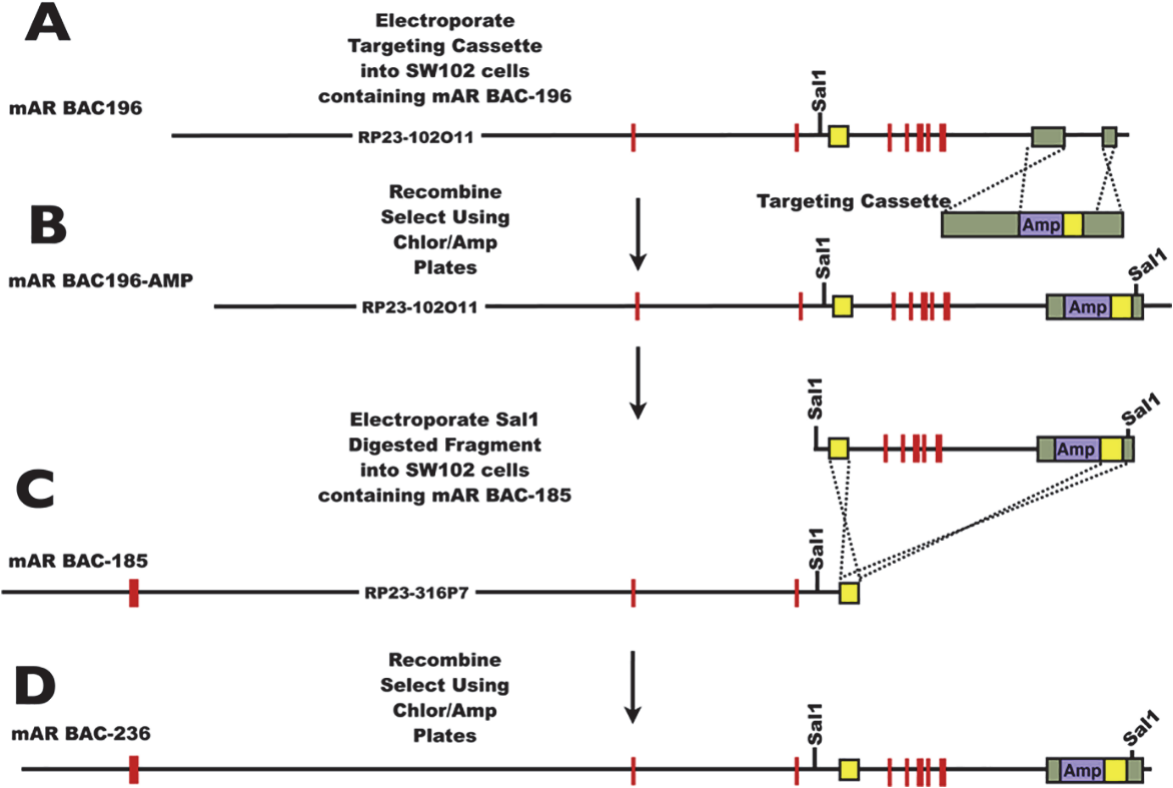
Construction of mAR BAC-236. Portions of the BACs mAR BAC-185 and mAR BAC-196 were combined to produce mAR BAC-236 that contained all AR exons and introns plus at least 40 kb each of 5’ and 3’ regions. (A) The targeting cassette derived from pAR2.6Amp (see S1 Fig.) was transformed into SW102 cells that contained mAR BAC-196. (B) The recombination produced mAR BAC-196Amp where a *SalI* site was introduced at the 3’ end just 3’ of a recombination arm common to both mAR BAC-185 and mAR BAC-196. (C) The 3’ *SalI* fragment from mAR BAC-196Amp was isolated and transformed into SW102 cells that contained mAR BAC-185. (D) Recombination of the 3’ *SalI* fragment with mAR BAC-185 added the extended 3’ sequence of mAR BAC-196 to mAR BAC-185 at the common recombination arm to produce the larger mAR BAC-236.

The mAR BAC-185 and mAR BAC-196 BACs, having the most extensive 5’ and 3’ sequences respectively of the three BACS, were linked together following techniques described by Sopher et al. [24], using a targeting cassette constructed from a PCR product (S1 Fig.) containing 1176 bp of 3’ sequence from mAR BAC-196 (Fig. 2). The 1176 bp PCR product had *NotI* and *XbaI* sites that were engineered into the primers so the amplicon could be directionally subcloned into the multiple cloning site of pBluescript at *NotI*/*XbaI* (S1 Table.). A 1220 bp cassette from pBluescript including the ampicillin resistance (amp^r^) gene was amplified using PCR with primers that had *AvrII*/*NotI* and *BamHI*/*EcoRI*/*ClaI* sites added and inserted into *AvrII* and *ClaI* sites of the targeting cassette replacing 383 bp of AR sequence between the *AvrII* and *ClaI* sites. This process produced 598 bp and 276 bp homologous arms for recombination on both sides of the amp^r^ cassette. Another 543 bp PCR fragment from the 3’ end of mAR BAC-185 (S1 Table) was subcloned adjacent and 3’ to the amp^r^ gene using the *BamHI* and *EcoRI* restriction sites to yield the shuttle vector pAR2.6Amp (S1 Fig.). A *SalI* site was introduced into the targeting cassette at the 3’ end of the fragment from mAR BAC-185 via a PCR reaction. This Sal1 site was used later to release the recombined product from mAR BAC-196.

The mAR BAC-196 was transformed into the SW102 strain of *E*. *coli*. This strain uses the prophage λ *exo*, *bet* and *gam* gene products that are under temperature sensitive repression, to perform homologous recombination. The insert from pAR2.6Amp was released using the *NotI* and *XbaI* sites incorporated from pBluescript II SK- and this insert was transformed into the SW102 strain containing mAR BAC-196 by electroporation and was grown at 42°C for 15 min. to allow for recombination. Recombination was selected for using chloramphenicol/ampicillin plates. DNA from recombined strains resistant to ampicillin was tested using PFGE and Southern blotting to ensure that the correct recombination had taken place and that there was no deletions, additions or rearrangements of the BAC.

The 3’ end of the recombined BAC (mAR BAC-196Amp, Fig. 2) was isolated using the incorporated *SalI* site and the endogenous *SalI* site located just 5’ of the recombination arm that is common to both mAR BAC-196 and mAR BAC-185. The *SalI* fragment was isolated using PFGE to separate the smaller recombination cassette from the remainder of the BAC followed by agarase digestion and dialysis on 0.05 μm VMWP membranes (Millipore Corporation, Billerica, MA) floated on distilled water. The recombination cassette DNA was then electroporated into SW102 cells containing mAR BAC-185 and recombined as above. The resulting mAR BAC-236 (236 kb) was assessed using PFGE and Southern blotting to ensure correct recombination occurred.

The final step of adding an IRES–EGFP cassette in the 3’ UTR just 3’ of the AR stop codon was performed by inserting a GalK cassette into this position using the SW102 strain containing the mAR BAC-236 [25] (S2 Fig.). The GalK cassette was modified using primers having homology with the Galk cassette plus 50 bp overhangs (S2 Table) that are homologous to the mAR 236-BAC insertion site. This PCR product was then transformed into the bacteria with mAR BAC-236 and selected for in M63 minimal media where Galk is necessary for survival (SW102 is GalK negative). A PCR product with the same 50 bp overhang (S2 Table) was used to amplify the IRES-EGFP cassette that was transformed in the SW102 strain with mAR BAC-236 (GalK). The correctly recombined BAC in which the IRES-EGFP cassette replaced the Galk cassette was selected for using GalK counter selection plates containing 0.2% 2-deoxy-galactose (DOG). Galactose kinase phosphorylates 2-deoxy-galactose and produces a toxic product (2-deoxy-galactose phosphate). The bacteria containing the final construct mAR BAC-236E was grown in LB medium with chloramphenicol and the BAC was purified using a double acetate extraction, followed by two rounds of CsCl gradient separation. The CsCl band was extracted with water saturated 1-butanol, ethanol precipitated and resuspended in TE buffer. The resuspended DNA was dialyzed using 25 mm Millipore 0.05 μm VMWP membranes (Millipore) against injection buffer. The DNA was assessed by PFGE for purity and to ensure that the DNA was not fragmented. The DNA was then sent to the Transgenic Core at University of Texas Southwestern Medical Center for pronuclear injection. Concentrations of DNA injected into the pronucleus were titrated to result in incorporation of low copy numbers of the AR-EGFP transgene as determined previously by Dr. Robert Hammer (Director, UT Southwestern, Transgenic Core). The resulting mAR-BAC-236E germ line transmitted transgenic founder mice are available upon request and have been placed in storage at the Jackson Laboratory (Bar Harbor, Maine).

### Animal care and use

Animals used in these studies were maintained and euthanized according to the principles and procedures described in the NIH Guide for the Care and Use of Laboratory Animals. Mice were housed in a temperature and humidity controlled facility with a 12 h light/dark cycle. AR^flox/flox^ mice were obtained from Guido Verhoeven [26]. AMHCre mice were purchased from JAX mice (Jackson Laboratory, Bar Harbor, ME). Mice were sacrificed by CO_2_ asphyxiation followed by cervical dislocation.

### Genotyping

PCR genotyping of mouse tails was used to identify mice containing the AR-EGFP transgene (3’EGFPfor and 3’EGFPrev primers, Table 1), Cre recombinase (CREfor, CRErev primers) and the AR gene containing flox sites in the introns surrounding exon 2 (AR exon 2 locus for, AR exon2 locus rev primers).

**Table 1:** Primers used for PCR genotyping.

Primer	Sequence
3’EGFP for	5’-AAAGACCCCAACGAGAAGCG-3’
3’EGFP rev	5’-TATCCATCTGCCCTCCTTGTCC-3’
CRE for	5’-GCATTACCGGTCGATGCAACGAGTG-3’
Cre rev	5’-GAACGCTAGAGCCTGTTTTGCACGTTC-3’
AR exon2 locus for	5’-AGCCTGTATACTCAGTTGGGG-3’
AR exon2 locus rev	5’-AATGCATCACATTAAGTTGATACC-3’

### Preparation of whole cell extracts and western blotting

To prepare whole cell lysates, mouse tissues from decapsulated testes, epididymis, heart, liver, kidney, muscle and brain were isolated and homogenized in enhanced lysis buffer (ELB, 250 mM NaCl, 0.1% NP40, 50 mM Hepes, pH 7.0, 5 mM EDTA, 0.5 mM dithiothreitol) with a protease inhibitor cocktail, rocked for 15 min at 4°C and then pelleted (12,000 x g 15 min) to remove cell debris. Cell lysates were fractionated by SDS-PAGE, transferred to polyvinylidene difluoride (PVDF) membranes, and incubated with polyclonal primary rabbit antibodies EGFP, (Cell Signaling #2956) at 1:1000, followed by horseradish peroxidase (HRP)-conjugated second antibody. The antigen-antibody complex was visualized with Millipore Immobilon^TM^ Western Chemiluminescent HRP substrate (Millipore). Immunoreactive signals from western blot films were scanned with an Epson 1600 Expressions scanner using Epson Scan software.

### Analysis of testis morphology, EGFP and AR expression

Testes were isolated and weighed followed by fixation in Bouin’s solution or 4% paraformaldehyde and parrafifin embedded. For analysis of testis morphology, Bouins fixed tissue sections (5 μm) were stained with a periodic acid-Schiff staining kit (Sigma, St. Louis, MO) followed by staining with hematoxylin. For analysis of EGFP or AR expression, paraformaldehyde fixed sections were probed with either primary goat antisera against GFP (Abcam Inc., Cambridge, MA ab5450, 1:1000 dilution) or polyclonal primary rabbit antisera against AR (Santa Cruz Biotechnology, Santa Cruz, CA AR C19, Sc-815, 1:1000 dilution). For EGFP imunodetection, biotin conjugated mouse anti-goat secondary antisera (Vectastain Elite ABC kit, Vector Laboratories, Burlingame, CA) was added and bound antibodies were detected as described by the kit instructions using DAB staining solution. Slides were counterstained with hematoxylin. For AR immunodetection, secondary donkey anti-rabbit antisera with conjugated cy3 fluor was added followed by staining with DAP1(4',6-diamidino-2-phenylindole). A charged coupled device (CCD) video camera system was used to capture images of tubule cross-sections.

### Isolation of genomic DNA from tissue and qPCR assays to determine transgene copy numbers

Genomic DNA was extracted from testis samples using the DNeasy Blood and Tissue kit (Qiagen Inc, Valencia CA) according to the manufacturer’s instructions with the following modification: an additional 15 min incubation with RNase (10 mg/mL) was included after tissue digestion in lysis buffer. DNA concentrations were obtained using spectrophotometric determination (OD_260_). Quantitative PCR (q-PCR) was performed on isolated testis genomic DNA using the Applied Biosystems 7500 Fast Real-Time System (Life Technologies Carlsbad CA) and SYBR Green PCR Master Mix (Life Technologies Carlsbad CA). For each reaction, 100 ng of genomic DNA and a final primer concentration of 250 nM each were used. Primers were designed to obtain amplicons of 212 or 245 bp. A common forward primer (Common AR For1) was used that anneals to the extreme 3’ end of the sequences flanking the AR gene that are present on the X-chromosome and in BAC-236E (S3 Table). Two reverse primers were used. One primer (Gen AR Rev1) anneals 212 bp downstream on the X chromosome, whereas the second reverse primer (Trans AR Rev2) is located within the ampicillin cassette that is present only at the 3’ end of BAC-236E. All reactions were 40 cycles using standard ABI cycling conditions (initial 2 min at 50°C, 10 min at 95°C, and 40 cycles of 15 s denaturation at 95°C and 1 min annealing and extension at 60°C). Mean C_t_ (cycle threshold) values were determined from triplicate samples in two separate experiments.

Prior to performing copy number assessments, dilutions of genomic DNA from mice containing endogenous AR sequences and BAC-236E were used to generate standard curves that provided the amplification efficiencies for the amplicons derived from endogenous flanking AR sequences and BAC-236E. The amplification efficiencies were found to be equivalent (within 0.5%). For each transgenic mouse strain, the relative number of copies of endogenous AR sequences and BAC-236E derived sequences were determined from their respective C_t_ (cycle threshold) using the E^ΔCt^ method, where E is the efficiency of the reaction and ΔC_t_ = C_t_ (target gene) − C_t_ (control gene) (see Bio-Rad user bulletin #5279 at http://www.bio-rad.com). Because the amplification efficiencies for the 2 PCR products are approximately equal, the number of copies of BAC sequences can be normalized to the number of copies of endogenous AR sequences, which are present in one copy in the genome. The calculated copy numbers were reported after rounding to the nearest whole number. This method of determining the copy number requires small amounts of DNA and is found to be accurate for low (<10) transgene copy numbers [27, 28].

### Statistical analysis

Results were analyzed by ANOVA with Newman-Keuls PLSD at a 5% significance level utilizing GraphPad Prism 4.3 (GraphPad Software, San Diego, CA).

## Results

### Construction of a BAC Containing the AR and EGFP Genes Flanked by 40 kb Each of AR 5’ and 3’ Sequences

Two bacterial artificial chromosomes (BACs) containing overlapping regions of the AR gene locus were recombined to create a new BAC denoted mAR BAC-236 that encompasses the entire 167 kb of the genomic mouse AR gene plus 40 kb each of 5’ and 3’ flanking sequence (Fig. 1). Specifically, the BACs mAR BAC-196 and mAR BAC-185 having the most extensive AR 5’ and 3’ flanking sequences were recombined via the use of a targeting cassette (S1 Fig.). A targeting cassette containing the 3’ end of mAR BAC-196 plus a region common to both BACs was used to add an ampicillin resistance gene and a *Sal1* restriction site to the 3’ end of mAR BAC-196. A *SalI* fragment consisting of the region extending from the second intron of AR through the 3’ end of mAR BAC-196 that included the targeting cassette was excised and recombined with mAR BAC-185 to produce mAR BAC-236 containing the entire AR gene plus extended 5’ and 3’ flanking sequences (Fig. 2). To facilitate the identification of cells expressing the AR transgene, a DNA cassette containing an internal ribosome entry site (IRES) allowing the bicistronic expression of EGFP from the same mRNA transcript was placed 3’ to AR stop codon in mAR BAC-236 to create mAR BAC-236E (S2 Fig.).

### Creation of Founder Mice Expressing the AR-GFP Transgene

**Fig 3:**
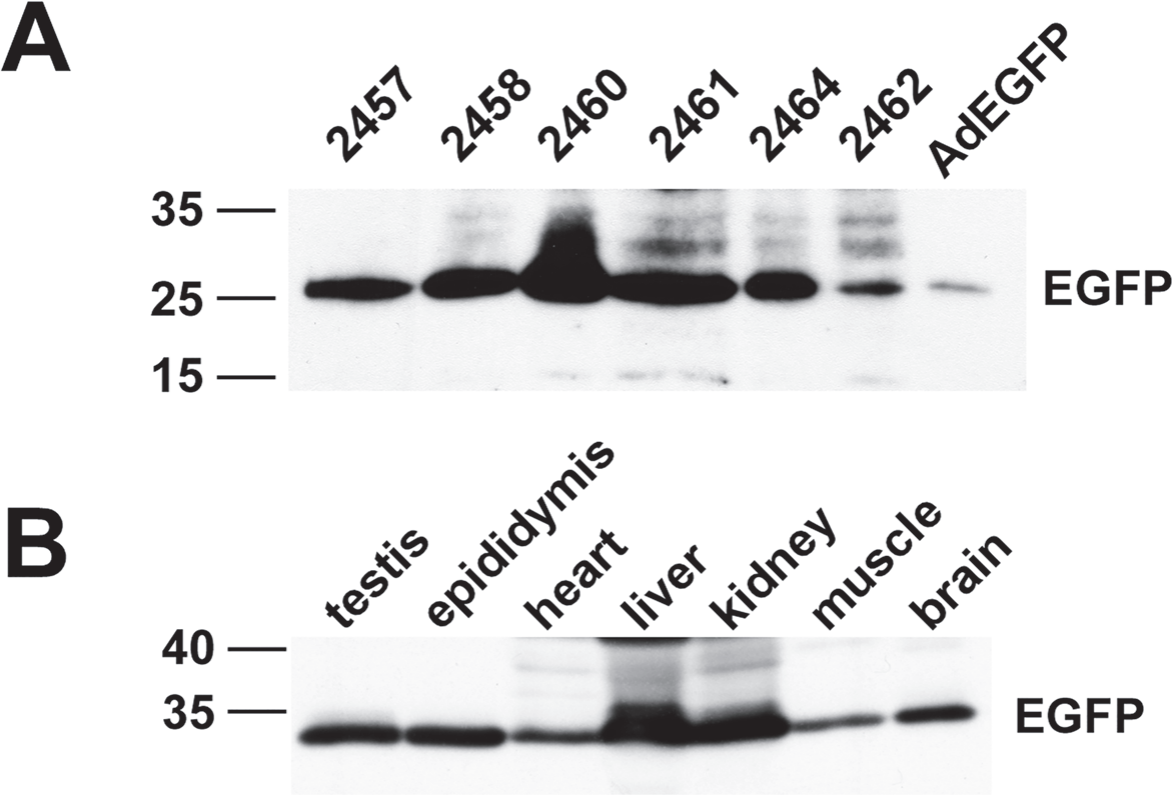
Expression of the AR-EGFP transgene in founder mice. A) Testis whole cell extracts from 6 founder mice having germ line transmission of the AR-GFP transgene and extracts from Cos7 cells infected with an adenovirus expressing EGFP (AdEGFP) by western blot. B) Whole cell extracts from the 2460 founder strain containing the AR-EGFP transgene were assayed for EGFP expression by western blot.

The mAR BAC-236E construct was used for pronuclear injection and 9 founder mice containing germ line transmitted mAR BAC-236E were obtained. Whole testis extracts were assessed by western blot for expression of EGFP as a marker for expression of the mAR transgene. These studies showed that six of the nine lines of transgenic mice expressed EGFP in the testis (Fig. 3A). EGFP also was expressed in other tissues that are testosterone responsive (Fig. 3B).

### The AR-GFP Transgene Compensates for the Loss of the Endogenous AR Gene in Sertoli Cells

**Fig 4:**
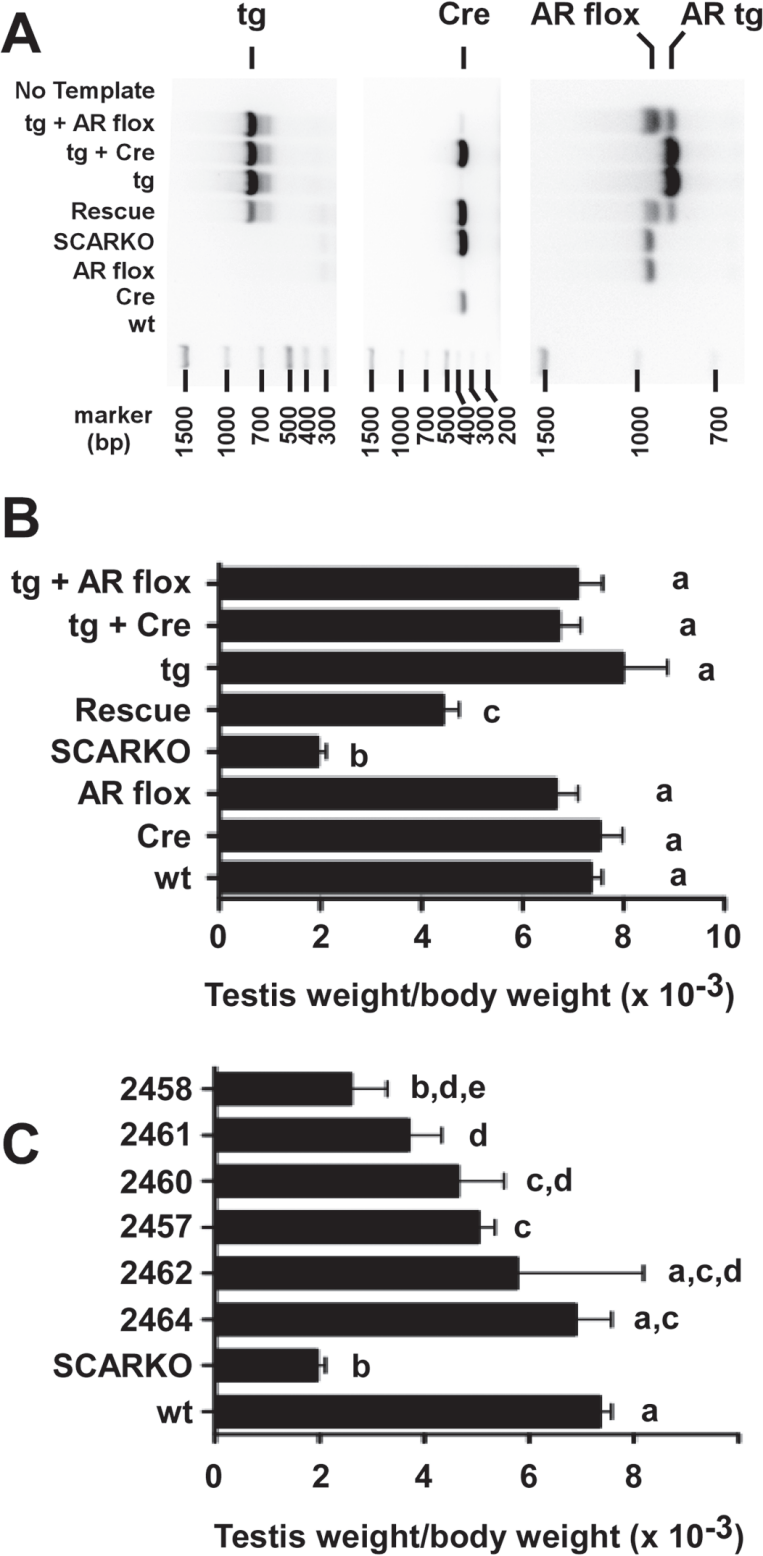
The AR-EGFP transgene increases testis weights in rescue mice. A) Agarose gel analysis is shown of PCR reactions used to genotype mice employed primers that identify the AR-EGFP transgene (tg), the Cre recombinase (Cre) or floxed endogenous AR (AR flox) and the AR transgene (AR tg). Mouse strains were identified that expressed no transgenes (wild type, wt), the Cre recombinase (Cre), a floxed AR gene (AR flox) as well as SCARKO and rescue mice. Mice expressing the AR-EGFP transgene (tg), the transgene + Cre (Tg + CRE) or the transgene in the presence of a floxed AR gene (Tg + AR flox) also were identified. The sizes of DNA markers in base pairs (bp) are shown below each gel image. B) Testis weight/body weights are provided for mice expressing the transgenes as described in A. C) The testis weight/body weight for the individual rescue mouse strains are shown relative to the same wt and SCARKO values used in panel B. Values with different lowercase letters differ significantly (P<0.05). Panel B, n = 6 to 39, panel C, n = 3 to 14).

The mouse lines that expressed the AR-EGFP transgene were mated with mice expressing the Cre recombinase driven by the Sertoli cell specific AMH promoter (AMH-Cre). The resulting male double transgenic mice (AMH-Cre; AR-EGFP/AR^+^/^y^) were mated with female mice in which either one or both alleles of AR had flox sites flanking exon 4 (AR^flox^ mice, obtained from Guido Verhoeven) [26]. Genotyping of the male offspring from these matings identified mice having only floxed AR, or floxed AR plus the Cre recombinase that should eliminate AR expression resulting in a SCARKO phenotype or the combination of floxed AR, Cre recombinase plus the AR-EGFP transgene in addition to 6 other genotypes (Fig. 4A). Adult testis weight (corrected for body weight) was used to determine the effect of each genotype. Mice having endogenous AR expression plus either Cre recombinase or floxed AR had testis weights similar to that of mice having no transgenes (wild type) (Fig. 4B). In contrast, testis weight was reduced to 26% of wild type in SCARKO mice in which both AMH-Cre and floxed AR were present. When the AR-GFP transgene was added to the SCARKO background, the transgene was able to rescue the SCARKO phenotype with a mean increase of testis weight to 65% of wild type for 6 founder strains. Incorporation of the AR-EGFP transgene alone, AR-EGFP transgene + AMH-Cre or AR-EGFP transgene + floxed AR did not alter testis size relative to wild type.

Individual AR-EGFP founder transgenic mouse strains were investigated further to determine the extent of the rescue phenotype. The AR-EGFP transgene strain 2464 permitted complete rescue of SCARKO mice with testis weights similar to that of its wild type siblings (Fig. 4C). Strains 2462, 2457, 2461, 2460 and 2458 showed varying levels of rescue with mean testis weights that were 78%, 68%, 63%, 50% and 35% of wild type, respectively. These data suggest that the AR-EGFP transgene provided either complete or partial rescue of spermatogenesis depending on the transgenic mouse strain. Expression of the AR-EGFP transgene in addition to endogenous AR did not affect testis weight.

### AR-GFP Transgene Expression in Sertoli Cells Matches That of Endogenous AR

**Fig 5:**
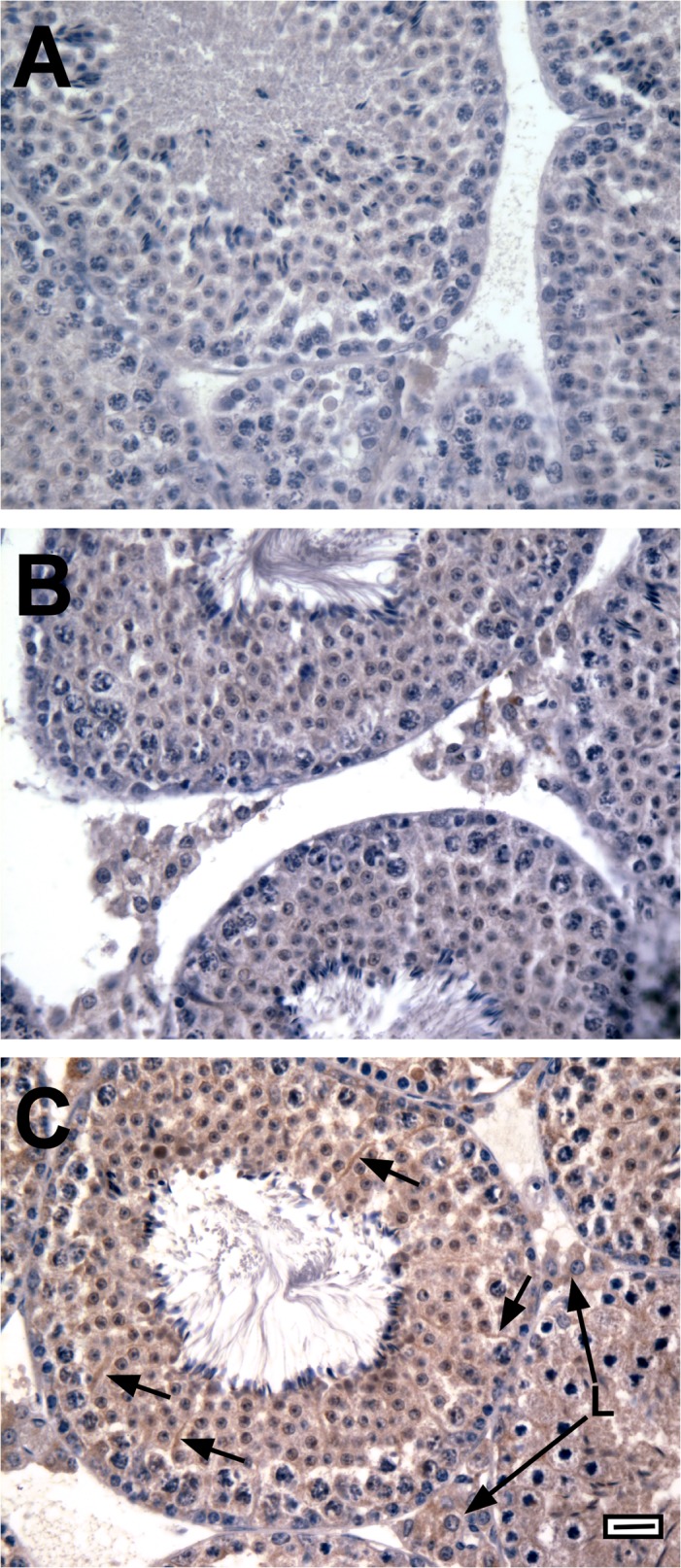
EGFP is expressed in the testes of mice containing the AR-EGFP transgene. Testis tissue sections from mice containing the AR-GFP transgene (A, C) and a mouse lacking the transgene (B) were probed with secondary antisera alone (A) or with antiserum against GFP and secondary antisera (B, C). Strong EGFP immunoreactivity (brown stain) was observed only in testes containing the AR-GFP transgene probed with EGFP antiserum (C). EGFP immunoreactivity was present in the cytoplasm of Sertoli cells including concentrations of EGFP in the Sertoli cell cytoplasm surrounding germ cells (arrows) and in the cytoplasm of Leydig cells (L, long arrows). Nuclei are stained blue with hematoxylin. Bar = 100 μm, magnification: 40x objective.

**Fig 6:**
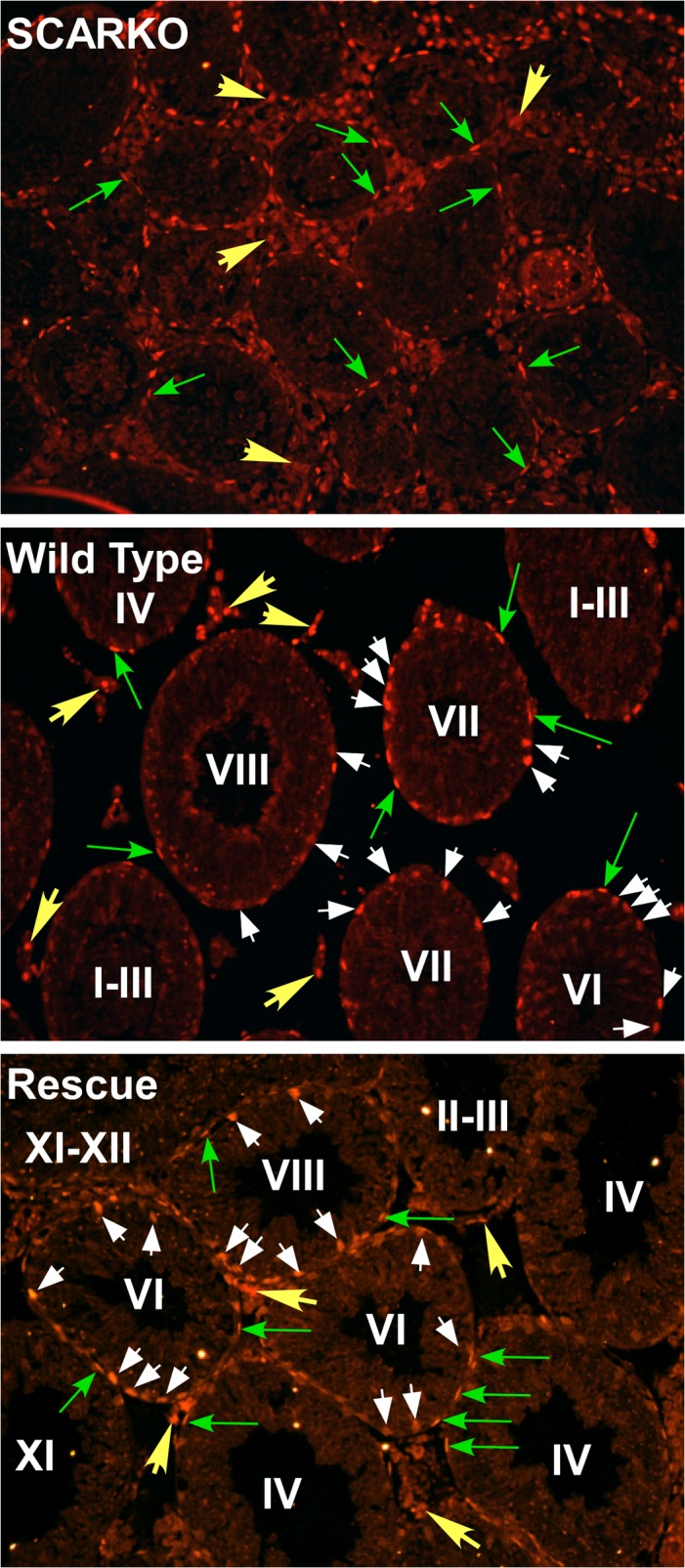
AR is expressed stage-specifically in Sertoli cells of rescue mice. Testis cross sections from SCARKO, wild type and rescue mice were probed with antisera against AR. Long green arrows denote AR immunostaining (red immunofluorescence) in peritubular cell nuclei. Short yellow arrows show AR expression in Leydig cells. White arrowheads denote AR immunostaining in Sertoli cell nuclei. Stages of the seminiferous epithelium for tubule cross sections are denoted by Roman numerals (I-XII). Bar = 100 μm, magnification: 20x objective.

To confirm that the AR-EGFP transgene was expressed in the testes of the transgenic mice in a cell-specific manner, adult testis tissue sections were probed for the expression of EGFP. In mice containing the AR-EGFP transgene, EGFP immunoreactivity was observed throughout the cytoplasm of Sertoli cells surrounding germ cells as well as in Leydig cells that are known to produce AR (Fig. 5). Immunofluorescence studies of AR expression also were performed on testis tissue sections from SCARKO, wild type and rescue mice. AR expression was not detected in Sertoli cells of SCARKO mice, as expected (Fig. 6). The SCARKO mice also had smaller seminiferous tubule diameters due to the lack of post meiotic germ cells. In wild type mice, AR immunoreactivity was detected in Sertoli cell nuclei in a stage-specific manner such that AR expression was detected in stage V-VIII seminiferous tubule cross sections with stages VI and VII having the most intense AR staining. Testes from rescue mice also displayed AR immunoreactivity in Sertoli cell nuclei in a stage-specific fashion mirroring that of the wild type mice. AR expression in peritubular cell and Leydig cell nuclei was similar for the SCARKO, wild type and rescue mice.

### The AR-GFP Transgene Restores Full Spermatogenesis to Testes Lacking AR in Sertoli Cells

**Fig 7:**
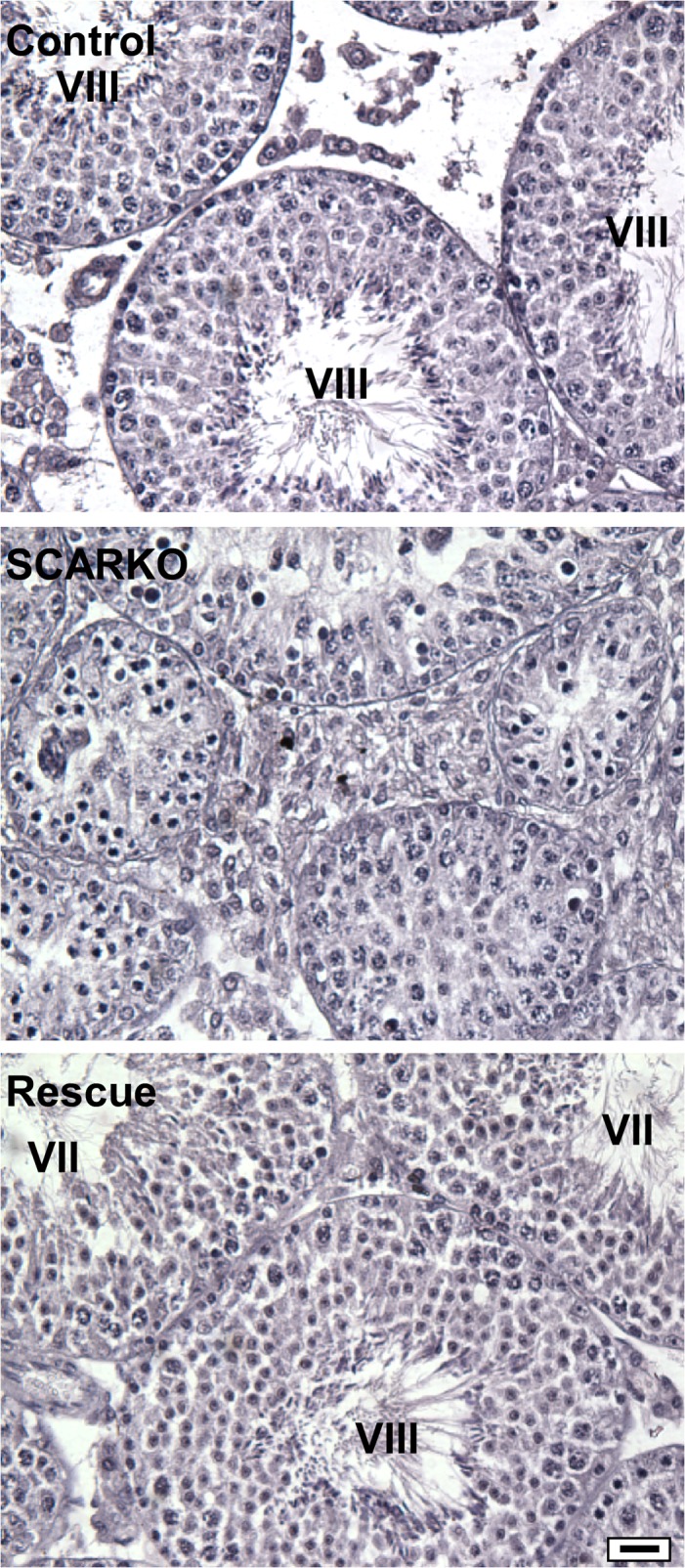
Expression of the AR-EGFP transgene rescues full spermatogenesis in a SCARKO background. Testes cross sections stained with PAS-Hematoxylin are shown for postnatal day 105 mouse littermates (founder 2464) that express only Cre recombinase (Control), Cre recombinase in the presence of a floxed AR gene (SCARKO), and a SCARKO mouse expressing the AR-EGFP transgene (Rescue). Bars = 100 μm, magnification: 40x objective.

As shown previously [26, 29], histological analysis of testis cross sections revealed that the diameters of seminiferous tubule cross sections were decreased and spermatogenesis was halted at the pachytene spermatocyte stage of meiosis in SCARKO mice (Fig. 7). In contrast, the expression of the AR-EGFP transgene in a SCARKO background (rescue mice) restored full spermatogenesis in 5 of the 6 transgenic lines as shown by the presence of round and elongated spermatids as well as mature spermatozoa poised to be released (Fig. 7 and Table 2). As shown previously [30], spermatogenesis proceeded normally in control mice containing either Cre recombinase (n = 7) or floxed endogenous AR (n = 8) only (data not shown). In agreement with the assays of testis weight, expression of the AR-EGFP transgene from strain 2464 permitted full spermatogenesis in nearly all seminiferous tubule cross sections (Fig. 7 and Table 2). Strains 2462, 2457, 2460 and 2461 displayed complete spermatogenesis in more than 50% of tubule cross-sections. Strain 2458 displayed partial spermatogenesis rescue allowing germ cells to proceed to an early stage of elongated spermatid development in about 10% of seminiferous tubules. The extent of spermatogenesis rescue (Table 2) and testis weight restoration (Fig. 4) in the presence of the AR-EGFP transgene were not correlated with the number of transgene copies integrated into the genome, which ranged from 1 to 3 copies in each strain (Table 2). In the rescued mice from the 5 strains that allowed completion of spermatogenesis, sperm were observed in the epididymis (data not shown). Furthermore, fertility was restored by the AR-EGFP transgene as the male rescue mice sired liters. Specifically, four male rescue mice derived from the 2457 founder strain were mated with seven wild type females. Pups were produced in six of eight matings with 3, 6, 8, 6, 3 and 10 pups per litter. These mice provide the first *in vivo* model in which an AR transgene is capable of rescuing AR function in a Sertoli cell specific AR null background. These results prove that the mAR BAC-236E construct has sufficient regulatory sequence to not only direct expression of AR in Sertoli cells *in vivo* but express AR in a stage specific manner so that spermatogenesis and fertility can be restored.

**Table 2:** Summary of spermatogenesis rescue by specific founder strains.

Line	Transgene copy number	Age at sacrifice (days)	# of mice tested	Rescue
2457	1	81–133	8	+++
2458	3	83–106	4	-
2460	3	86, 105	2	+++
2461	3	73–114	3	+++
2462	3	78–116	2	+++
2464	2	43–101	3	++++

No spermatids present

+ A few elongated spermatids present

++ Complete spermatogenesis in >10% of tubule cross sections

+++ Complete spermatogenesis in >50% of tubule cross sections

++++ Complete spermatogenesis in >90% of tubule cross sections

## Discussion

We report the first transgenic rescue of AR function. We obtained 9 founder mice from our pronuclear injections, of which 6, expressed EGFP from the AR-EGFP modified BAC transgene. This number of successful founders suggest that the transgene is insulated from copy number and position effects due to its size. Because the AR gene is required for fertility and is hemizygous (present only on the X chromosome) in males, the crosses required to produce rescue mice in males were made more complex. Nevertheless, we found that expression of the AR-EGFP transgene from 5 of the founders was capable of restoring spermatogenesis in SCARKO mice that lack Sertoli cell-specific endogenous AR expression. The rescue of testis size and sperm production varied for the 5 mouse strains. One founder strain was found to rescue testis size to normal levels and reestablished full spermatogenesis in nearly all seminiferous tubule cross sections. Other strains were capable of restoring full spermatogenesis but not in all tubules. The mechanisms responsible for the variable rescue of spermatogenesis are not yet understood. However, it is possible that the extent of rescued spermatogenesis may be related to the expression levels of the AR-EGFP transgene and the capability of each mouse strain to express the transgene in Sertoli cells in a stage specific manner. In wild type rodents, AR is expressed at high levels during stages VI-VIII during the cycle of the seminiferous epithelium but AR expression is difficult to detect during other stages [31]. Also, it is possible that transgene positional effects in some of the strains may limit transgene expression or disrupt the stage-specific expression of AR. Unlike most cases of BAC transgenesis, where the size of gene flanking regions is very large, the mARBAC-236 flanking regions were limited to about 40 kb each due to the size of the AR gene encompassing most of the BAC. Nevertheless, the rescue of the SCARKO phenotype such that complete spermatogenesis and sperm production are restored after the introduction of the AR-GFP transgene indicates the transgene was successful in replicating stage-specific AR expression supporting spermatogenesis.

The AR-EGFP transgene was able to restore male fertility in otherwise infertile SCARKO mice as evidenced by the pups produced by the mating of mice derived from the 2457 founder strain in the SCARKO background. However, pups were not produced in 2 of 8 matings and small litter sizes were observed for two other matings. The lower fecundity of some of the matings may be explained by the intermediate rescue phenotype displayed by the 2457 strain that displayed a testis size that was restored to only 68% of wild type. Follow up studies will include evaluation of the 2464 line of rescue mice to assess the fertility of mice that had testis size and spermatogenesis restored to wild type levels.

There have been no previous reports of transgenic animals that successfully express AR using limited 5’ regulatory sequences. The use of larger transgenes for AR was first reported by Sopher et al. in which a 450 kb YAC containing the 100 kb human AR gene was modified by homologous recombination to add 100 CAG repeats to exon 1 of the human AR gene. This AR transgene successfully recapitulated X-linked spinal and bulbar muscular atrophy (SBMA) in mice [32]. This approach was repeated using a BAC in which the same group added 162 CAG repeats to the human gene. For this construct, the two BACs were combined using a recombination strategy that employed antibiotic positive selection and the two-step recombineering strategy that we emulated [24]. However, we then used a Galk negative selection pop in/pop out strategy to insert an IRES.EGFP cassette [33].

Further evidence that substantial 5’ regulatory sequences are required for correct AR expression was provided by Ye et al. [34]. These investigators produced a transgenic mouse using a BAC (BAC-192, Fig. 1) having 15.2 kb of AR upstream sequence including all AR gene exons and introns plus 10 kb of downstream sequence. The BAC also encoded either the LacZ/EGFP or Luciferase gene regulated by promoters under the control of AR to assay AR expression from the BAC. Using this construct, it was found that expression of the AR transgene was directed to Sertoli cells in the testis and that the expression was strongest in stages VII and VIII, which is characteristic of endogenous AR expression [31]. In comparison, our transgene contained an additional 25 kb of 5’ upstream sequence and 30 kb of downstream sequence. The BAC was injected as circular construct. Based on previous studies, we expect these constructs to roll out in tandem copies such that at least one full-length copy of the transgene is incorporated into the genome with contiguous flanking sequences [35–37].

The importance of correct temporal and cell-specific expression of AR transgenes was shown previously using a model in which expression of an AR transgene was driven by the Sertoli cell-specific Androgen Binding Protein (ABP) promoter in the presence of an intact endogenous AR gene [38]. In this transgenic model, testis size was reduced to 60% of normal. This gain of function transgenic mouse was found to strongly express the AR transgene in Sertoli cells at early stages of development in neonates. The use of the heterologous ABP promoter caused elevated levels of AR in Sertoli cells prior the normal onset of AR expression, thus causing the immature Sertoli cells to cease proliferating and differentiate earlier during testicular differentiation. The precocious expression of AR resulted in fewer Sertoli cells allowing fewer germ cells to be supported. In contrast, we did not observe any alteration in testis size in mice retaining endogenous AR expression plus the AR-EGFP transgene. This result suggests that the expression of the AR-EGFP transgene does not initiate prior to endogenous AR expression and thus does not interfere with normal Sertoli cell proliferation and developmental patterns. Also, we did not observe any delay in the initiation of spermatogenesis (data not shown). Thus, AR-EGFP transgene expression in Sertoli cells appears to initiate at approximately the same developmental time point as endogenous AR, although assays of AR-EGFP transgene expression through development must be performed to confirm this idea.

In the adult testis, expression of the AR-EGFP transgene is confined to the seminiferous tubule stages where endogenous AR is expressed. The restriction of AR-EGFP transgene expression to stage VI-VII Sertoli cells provides additional evidence that the transgene is expressed under tight regulation in a manner similar to that of endogenous AR. This timing and proper regulation of AR expression in the transgenic rescue mice is likely due to the use of the extensive genomic AR transgene and its intronic and associated 5’ and 3’ flanking regulatory regions (40 kb each). The lack of any effect on testis size in the presence endogenous AR expression plus the AR-EGFP transgene also suggests that expression of the AR-EGFP transgene is not affected by copy number and may be under strict physiological feedback control due to the large AR flanking regions that are associated with the AR-EGFP transgene. This proposed feedback control of AR transgene expression may explain the similar levels of spermatogenesis rescue that occur in the presence of 1 to 3 copies of the transgene. The proper expression profile of the AR transgene in Sertoli cells suggests that the transgene mimics the endogenous AR gene in other AR expressing tissues. To this end, the gross morphological structure of the Leydig cells and peritubular cells of the testis appeared normal, which suggests again that AR-EGFP transgene did not affect endogenous AR function in these cells. Finally, normal AR male mice with the transgene were functionally fertile and did not show any abnormalities.

The purpose of producing this mouse model expressing the AR-EGFP transgene was to provide proof of principle that the functional domains of AR protein could be studied through *in vivo* mutational analysis in the context of androgen signaling in the testis. Using the strategy that we have validated, additional transgenic mouse strains can be created in which endogenous AR is replaced with mutated AR constructs that can selectively activate only the classical or non-classical signaling pathway. In the future, these transgenic models could be used to study the functions of each testosterone-mediated pathway that are required to maintain spermatogenesis. These transgenic mice also may allow the identification of molecular and cellular mechanisms by which testosterone acts in Sertoli cells to maintain fertility and to facilitate the design of therapies to cure specific defects in testosterone-mediated signaling that cause infertility. Furthermore, transgenic mice incorporating the AR transgene or mutated versions of the AR transgene could be used to test male contraceptive candidates based on the blocking of specific testosterone dependent processes during spermatogenesis. Finally, the AR transgene and mutated derivatives may be used to study testosterone regulation of physiologically important processes in other tissues including oocyte development, sexual differentiation, heart function, muscle mass maintenance and prostate tumor progression.

## Supporting Information

S1 Fig Strategy used to construct the targeting cassette pAR2.6-AMP for the addition of the 3’ flanking region of the AR gene onto mAR BAC-185. A 1176 bp *NotI*-*XbaI* fragment at the 3' end of the mAR BAC-196 was amplified using PCR. A 383 bp *AvrII*-*ClaI* fragment was removed by restriction digestion and a 1220 bp PCR product containing the amp^r^ cassette with flanking *AvrII* and *Cla1* sites from pBluescript SK- was inserted into the 1176 bp fragment. *BamHI* and *EcoRI* sites were introduced into the amp cassette PCR product. The recombination arm (denoted B, common to both mar BAC-196 and mar-185) was amplified by PCR and inserted into the *BamHI* and *EcoRI* sites. The amplification of the recombination arm introduced a *SalI* site into the cassette allowing the removal of the 3’ *SalI* fragment in a later step. The final targeting cassette was named pAR2.6-Amp.
(PDF)

S2 Fig Creation of the bicistroninc AR-IRES-EGFP transgene. The Galk cassette was amplified using primers with 50 bp overhangs that were homologous to regions within exon 8 of AR This GalK cassette was selected for using minimal media plates containing galactose. The IRES-EGFP cassette was amplified by PCR using primers having the same 50 bp overhangs. Selection for BACs in which the Galk cassette was replaced by the IRES-EGFP cassette was performed on 2 deoxy galactose (DOG) plates (negative selection for GalK).
(PDF)

S1 Table Oligonucleotides used to amplify the 543 bp fragment used for recombineering mAR BAC-185 to mAR BAC-196 to produce mAR BAC-236. All oligonucleotides are 5’ to 3’.
(PDF)

S2 Table Oligonucleotides used to produce PCR products to introduce the GalK cassette into a position immediately 3’ to the stop codon of AR in Exon 8. Underlined sequences are GalK or IRES.EGFP specific whereas other sequences are AR specific. The GalK cassette was then replaced with an IRES.EGFP cassette that did not have a PolyA sequence so that the mRNA produced would use the polyadenylation signal of AR. This recombineering step produced mAR BAC-236E. All oligonucleotides are 5’ to 3’.
(PDF)

S3 Table Primers used for qPCR copy number evaluation. (PDF)
