# Supplementary material for: Restoration of Spermatogenesis and Male Fertility Using an Androgen Receptor Transgene
Source: PLoS One. 2015 Mar 24;10(3):e0120783. doi: 10.1371/journal.pone.0120783 (PMC4372537; doi:10.1371/journal.pone.0120783)
Supplement: S1 Fig — A 1176 bp NotI-XbaI fragment at the 3' end of the mAR BAC-196 was amplified using PCR. A 383 bp AvrII-ClaI fragment was removed by restriction digestion and a 1220 bp PCR product containing the ampr cassette with flanking AvrII and Cla1 sites from pBluescript SK- was inserted into the 1176 bp fragment. BamHI and EcoRI sites were introduced into the amp cassette PCR product. The recombination arm (denoted B, common to both mar BAC-196 and mar-185) was amplified by PCR and inserted into the BamHI and EcoRI sites. The amplification of the recombination arm introduced a SalI site into the cassette allowing the removal of the 3’ SalI fragment in a later step. The final targeting cassette was named pAR2.6-Amp. (PDF) [file pone.0120783.s001.pdf]

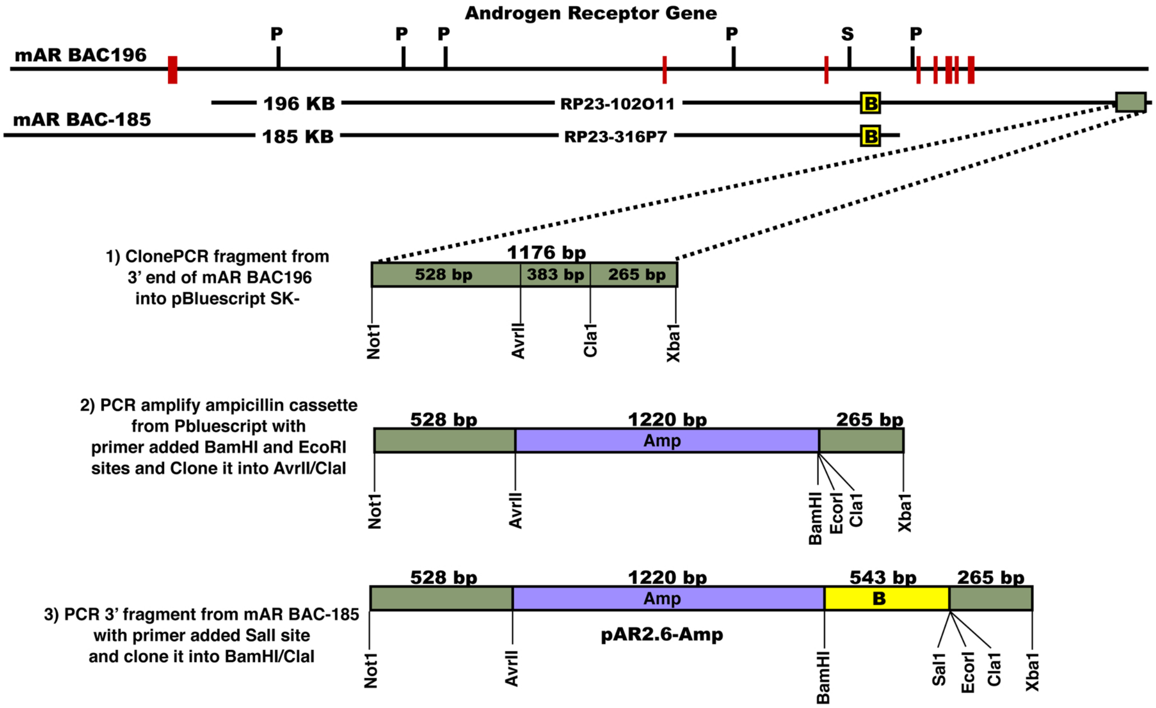
Androgen Receptor Gene
P
P
P
P
S
P
mAR BAC196
196 KB
RP23-102O11
B
mAR BAC-185
185 KB
RP23-316P7
B
1) ClonePCR fragment from
3' end of mAR BAC196
into pBluescript SK-
1176 bp
528 bp
383 bp
265 bp
Not1
AvrII
Cla1
Xba1
2) PCR amplify ampicillin cassette
from Pbluescript with
primer added BamHI and EcoRI
sites and Clone it into AvrII/ClaI
528 bp
1220 bp
265 bp
Amp
Not1
AvrII
BamHI
EcorI
Cla1
Xba1
3) PCR 3' fragment from mAR BAC-185
with primer added SalI site
and clone it into BamHI/ClaI
528 bp
1220 bp
543 bp
265 bp
Amp
B
pAR2.6-Amp
Not1
AvrII
BamHI
Sal1
EcorI
Cla1
Xba1
