# Supplementary figures and images for: Restoration of Spermatogenesis and Male Fertility Using an Androgen Receptor Transgene
Source: PLoS One. 2015 Mar 24;10(3):e0120783. doi: 10.1371/journal.pone.0120783 (PMC4372537; doi:10.1371/journal.pone.0120783)

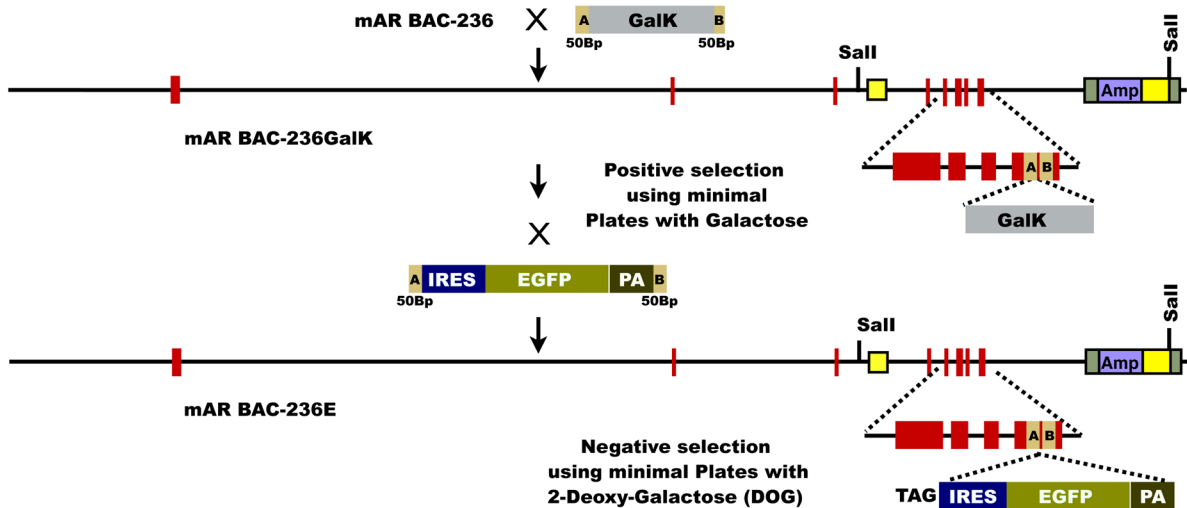

Supplement: S2 Fig — The Galk cassette was amplified using primers with 50 bp overhangs that were homologous to regions within exon 8 of AR This GalK cassette was selected for using minimal media plates containing galactose. The IRES-EGFP cassette was amplified by PCR using primers having the same 50 bp overhangs. Selection for BACs in which the Galk cassette was replaced by the IRES-EGFP cassette was performed on 2 deoxy galactose (DOG) plates (negative selection for GalK). (PDF) [file pone.0120783.s002.pdf]
