# Supplementary material for: Restoration of Spermatogenesis and Male Fertility Using an Androgen Receptor Transgene
Source: PLoS One. 2015 Mar 24;10(3):e0120783. doi: 10.1371/journal.pone.0120783 (PMC4372537; doi:10.1371/journal.pone.0120783)
Supplement: S1 Table — All oligonucleotides are 5’ to 3’. (PDF) [file pone.0120783.s003.pdf]

Primer Name	Oligonucleotide
1176 bp PCR-For	CAATATCCTCCTCAGCACCTCATC
1176 bp PCR-Rev	AGGCATCAAAATAAACGTACACAGG
543 bp PCR-For	TCCAAAATGGCAACCACCCC
543 bp PCR -Rev	AAACACAGGAGGCACGCAATGC
