# Supplementary material for: Restoration of Spermatogenesis and Male Fertility Using an Androgen Receptor Transgene
Source: PLoS One. 2015 Mar 24;10(3):e0120783. doi: 10.1371/journal.pone.0120783 (PMC4372537; doi:10.1371/journal.pone.0120783)
Supplement: S2 Table — Underlined sequences are GalK or IRES.EGFP specific whereas other sequences are AR specific. The GalK cassette was then replaced with an IRES.EGFP cassette that did not have a PolyA sequence so that the mRNA produced would use the polyadenylation signal of AR. This recombineering step produced mAR BAC-236E. All oligonucleotides are 5’ to 3’. (PDF) [file pone.0120783.s004.pdf]

| Primer Name | Oligonucleotide |
|---|---|
| Common AR For1 | 5’-GTGAGCGTGGACTTTCCTGA-3’ |
| Gen AR Rev1 | 5’- GAGGAATTTCCCCCAAGGCA-3’ |
| Trans AR Rev2 | 5’-CAAGAAGACAGGGCCAGGTT-3’ |
